# Reviews and Bibliographical Notices

**Published:** 1882-06

**Authors:** 


### REVIEWS AND BIBLIOGRAPHICAL NOTICES.

DISEASES OF WOMEN: Clinical Lectures, by Lombe Atthill, M.D., etc. Fifth edition. Philadelphia: P. Blakiston, Son & Co. Cloth, $1.25, Paper, 75c.

The above is one of a series of "*Handbooks for Physicians*" now being published by Messrs. P. Blakiston, Son & Co., at the uniform price of $1.25 in cloth and seventy-five cents in paper covers. The series includes some of the most practical manuals recently published. In addition to the work of Dr. Atthill, Dr. Habershon's excellent book on the "Diseases of the Stomach," Black on the "Functional Diseases of the Renal, Urinary, and Reproductive Organs," Sewell's "Dental Anatomy and Surgery," Heath's "Surgical Diagnosis," Fenwick's "Outlines of Practice" and a number of others are included in the series.

Any of the volumes can be bought separately at the price mentioned, and if five volumes or more are taken at a time, a considerable reduction is made in the price.

Atthill's Clinical Lectures on the Diseases of Woman are marked by strong common sense, a clear way of stating points of differential diagnosis and generally sound and judicious views upon treatment. The student or practitioner who follows him in his gynæcological practice will have a safe guide, and will rarely fail to benefit his patient. The book deserves a large circulation.

We cannot abstain from calling attention to the excellent manner in which the publisher's work is done. The paper, type, and presswork compare favorably with many high-priced books on the bookseller's shelves.

We trust the publishers will be encouraged to continue this excellent series of hand-books.

MANUAL OF DENTAL SURGERY AND PATHOLOGY; by Alfred Coleman, L.R.C.P., F.R.C.S., Exam. L.D.S., etc.; senior Dental Surgeon and Lecturer on Dental Surgery to St. Bartholomew's Hospital and to the Dental Hospital of London; member of Board of Examiners in Dental Surgery Royal College of Surgeons; formerly President Odontological Society of Great Britain. Thoroughly revised and adapted to the use of American students and practitioners, by Thos. C. Stellwagen, M.A., M.D., D.D.S., Prof. of Physiology at Phila. Dental College. Published by Henry C. Lea's Sons & Co., Phila. 408 pages, 400 illustrations.

Prof. Stellwagen has added about one hundred pages of valuable matter and one hundred and twelve illustrations to Prof. Coleman's *most excellent* work. This American edition brings dental surgery, with its many rapid strides in this specialty of medicine, in an easy and comprehensive style up to the present time. The profession is largely indebted to Profs. Coleman and Stellwagen for this invaluable book. The publishers, too, have handsomely sustained their reputation.

*Seventeenth Annual Announcement of the Missouri Dental College*—Session of 1882–3. Pp. 8.

The regular course of lectures will commence on September 23, 1882, and continue until March 1, 1883.

*The Massachusetts Eclectic Medical Journal* is a new addition to our list of exchanges. It presents a neat appearance, and is filled with judicious selections from the best regular medical journals.

*Working Bulletin for the Scientific Investigation of Jamaica Dogwood, Quebracho, and Cascara Sagrada.*—Detroit: Parke, Davis & Co.

These pamphlets give the history, botanical character, physical and chemical properties, physiological action and therapeutic application of the drugs mentioned. They are accompanied by plates and diagrams, and all facts known are given in a succinct and available manner, so that any one so disposed can continue the investigation of their value understandingly.

*The American Newspaper Directory* for 1882, published by Geo. P. Rowell and Co. of New York, has been received. It contains the names of 10,611 periodicals in the United States and Territories, which is a gain of 344 in the year just passed. The number of daily papers has increased in a somewhat larger proportion, and is now represented by a total of 996 against 921 in 1881. The largest increase has been in New York—10 dailies, 29 of all sorts. Illinois and Missouri show a percentage of gain which is even greater, while Colorado leads all others in the percentage of increase, both of daily and weekly issues. California, Nebraska, Nevada, Oregon, South Carolina, Tennessee, Vermont, and West Virginia have fallen behind 1881 in the total number of periodicals issued. In Georgia, Maine, and Massachusetts the suspensions have exactly counterbalanced the new ventures. In every State not mentioned above, and in the Territories, there has been an increase.

